# Mental health of immigrants from the former Soviet Bloc: a future problem for primary health care in the enlarged European Union? A cross-sectional study

**DOI:** 10.1186/1471-2458-7-27

**Published:** 2007-02-28

**Authors:** Yulia Blomstedt, Sven-Erik Johansson, Jan Sundquist

**Affiliations:** 1 Karolinska Institute, Center for Family and Community Medicine, Alfred Nobels allé 12; SE141 83 Huddinge, Sweden

## Abstract

**Background:**

Enlargement of the European Union has caused worries about the possibility of increased migration from its new members, the former Soviet countries, and consequently increased demands on the health care systems of the host countries. This study investigated whether or not earlier immigrants from the former Soviet Bloc have poorer self-reported mental health, measured as self-reported psychiatric illness and psychosomatic complaints, than the host population in Sweden. It also examined the particular factors which might determine the self-reported mental health of these immigrants.

**Methods:**

The cross-sectional national sample included 25–84-year-old Swedish-born persons (n = 35,459) and immigrants from Poland (n = 161), other East European countries (n = 164), and the former Soviet Union (n = 60) who arrived in Sweden after 1944 and were interviewed during 1994–2001. Unconditional multivariate logistic regression was used in the analyses.

**Results:**

The findings indicated that the country of birth had a profound influence on self-reported mental health. Polish and other East European immigrants in general had a twofold higher odds ratio of reporting psychiatric illness and psychosomatic complaints, which fact could not be explained by adjustments for the demographic and socioeconomic variables. However, immigrants from the former Soviet Union had odds similar to those of the Swedish-born reference group. Adjustments for migration-related variables (language spoken at home and years in Sweden) changed the association between the country of birth and the outcomes only to a limited extent.

**Conclusion:**

Since poor mental health may hinder acculturation, the mental health of immigrants from Poland and other East European countries should be acknowledged, particularly with the expansion of the European Union and inclusion of nine former Soviet Bloc countries by 2007.

## Background

Sixteen years after its dissolution, the Soviet Bloc started to be mentioned again in the media when seven of its former members (Poland, Hungary, Slovakia, the Czech Republic, Estonia, Latvia, and Lithuania) were invited to join the European Union (EU) in 2004, and another two (Bulgaria and Romania) in 2007. It is just as incorrect now to call these countries former members of the Soviet Bloc as to call the United States a former British colony [1]. However, the reasons for making this mistake are easy to explain. The isolation from the West by half a century of war and communist rule, the Iron Curtain, and the forced inclusion in the Soviet Bloc with the compulsory creation of the "Soviet-style regimes" [2] have all had a profound influence on the economy, social fabric, and health of the population in these countries and, although diminishing, the consequences are still visible. The former Soviet Bloc countries are worse off economically and the health rates there are lower than in the Western Europe [3]. Consequently, migration from these countries may cause a change in service needs and/or health patterns in the host societies. To prevent or manage such a change successfully, it is important to learn about the health of this immigrant group. The present study attempted to procure knowledge about the self-reported mental health of immigrants from the former Soviet Bloc currently living in Sweden.

Self-reported mental health (defined here as self-reported psychiatric illness and psychosomatic complaints) was the focus of this study because mental health is fundamentally interconnected with physical and social functioning and health outcomes and is therefore crucial to the overall well-being of individuals, societies, and countries [4]. Moreover, in general, self-reported health is a reliable indicator of the health status of a population, predicting, as it does, mortality, morbidity, and other clinical outcomes [5,6].

### Migration to Sweden from the Soviet Bloc to Sweden after 1944

Sweden, at the crossroads between the two sides of the Iron Curtain, has become not only one of several transit countries, but also a place of residence for numerous immigrants from Eastern Europe and the former Soviet Union (FSU) after the dissolution of the Soviet Bloc in 1989 and the FSU in 1991, with increasing migration from east to west. Even before that, refugees sought asylum in Sweden after revolts and political persecutions in the Soviet Bloc. However, due to the Cold War and the Iron Curtain, which significantly reduced European East-West migration, for a long time migration from countries of the former Soviet Bloc to Sweden was characterized by distinct 'waves' of migration directly linked to political events in the countries of emigration [7]. Some of the major events are presented in Table 1. However, the immigration politics of Sweden has also influenced this migration. For example, in the mid-70s, migration to Sweden was made easier for Polish-born individuals by the abolition of the necessity to obtain visas [8]. Sweden also started to recruit workers from Hungary in 1947.

**Table 1:** Events in the Soviet Bloc* and Migration to Sweden.

**Period**	**The basic dates in the history of the Soviet Bloc**	**Major events in the member countries which caused immigration waves in Sweden **[7, 8, 36, 67–70]
1945–49	1944–45: Occupation of Hungary by the Soviet Army 1945–47: Communists established as dominant force in Romania 1947: Proclamation of People's Republic of Bulgaria 1948: Communists take power, Czechoslovakia becomes a Soviet-style communistic state 1948: Romania becomes a Soviet-style communistic state 1949: Proclamation of the People's Republic of Hungary	1946–50 – Suppression of the anti-Soviet guerrilla movements in the Baltic republics of the Soviet Union (emigration to escape the Red Army) 1948–53 – Intensification of terror and anti-Semitic campaigns in all member countries of the Soviet Bloc, especially in the Soviet Union
1950–59	1952: Proclamation of People's Republic of Poland 1955: Signing of the Warsaw Pact (all countries)	1956 – Soviet military invasion of Hungary in response to revolution
1960–69		1968 – Soviet invasion of Czechoslovakia in response to the reforms of the "Prague Spring" 1967–73 – Anti-Semitic campaign in Poland 1968 – Start of the Polish political crisis (major student and intellectual protests against communist government) and consequent start of political persecutions in Poland
1970–79		The whole decade – economic difficulties, social discontent, strikes, protests, and consequent large-scale economic emigration from Poland
1980–89	1989 – Dissolution of the Soviet Bloc	1981 – The military coup in Poland (communist government against "Solidarity" and the strikers) 1982–1983 – Imposition of martial law in Poland: reinforced persecutions of members of democratic movements (formal banning of "Solidarity") and harder military control 1989 – Fall of the Iron Curtain and disappearance of the migration restrictions in Eastern Europe
1990–99	1991 – Breakdown of the Soviet Union	1991 – Disappearance of the migration restrictions in the former Soviet Union

* Only a brief list of basic dates and major events in the countries relevant for this study, not a complete chronicle of the Soviet Bloc's history

By 2001 there were about 110,000 immigrants from the former Soviet Bloc in Sweden. Table 2 illustrates the arrival patterns of these immigrants, which correspond to occurrences of major political events in these countries. Many of the immigrants from Hungary and the former Czechoslovakia arrived before 1969, i.e. in response to, respectively, the revolution in 1956 and the so-called "Prague Spring" in 1968. The arrival of Poles in Sweden dates back mostly to the 1970s, with an increase after the military coup in 1981. It was virtually impossible for inhabitants of the FSU to migrate before the breakdown of the empire and abolition of migration restrictions which resulted in the arrival of a wave of immigrants from the various former republics in Sweden after 1991.

**Table 2:** Immigrants from the Former Soviet Bloc in Sweden [71]

Country of birth	Total number of immigrants for the year indicated (persons)		Arrival of immigrants (number of persons per arrival period)**			
	1950*	2001	-1969	1970–79	1980–89	1990–99
Bulgaria	44	3,605	274	313	632	2,094
Former Czechoslovakia	3,548	7,835	3,196	1,551	1,376	1,086
Hungary	2,030	14,027	5,610	2,750	3,108	1,705
Romania	531	11,954	443	666	5,262	4,818
Poland	7,832	40,506	3,359	9,498	15,487	9,734
The former Soviet Union	37,997	30,504	4,726	1,092	1,855	12,173

* Mostly refugees of the World War II, many of whom have repatriated to their countries of birth later

** Only the arrival of immigrants who still lived in Sweden in 2001 (i.e. at the end of the study) is shown (not the arrival of the total number of refugees and immigrants during these periods).

### Health determinants among immigrants from the countries of the former Soviet Bloc. A review of the literature

Immigrants from the former Soviet Bloc have not been systematically studied as a group and previous studies have mostly focused on immigrants from the FSU. However, the determinants of mental health reported by these studies generally correspond with the characteristics which are known to determine the health (in terms of mortality, morbidity, or self-reported health) of the population in various countries of the former Soviet Bloc and which, we assume, can be associated with mental health among immigrants from these countries as well.

*Age *[9], *sex *[10,11], and *marital status *[12,13] have often been associated with the mental health of immigrants from the FSU.

Social capital is another widely-discussed health determinant. In Russia, and supposedly in other former Soviet Bloc countries [14], social capital takes the form of informal social networks, such as the family, relatives and friends, rather than formal institutions [15]. Studies in the countries of the former Soviet Bloc and on immigrants from these countries show that the support received from friends [16,17], emotional support in general [18], and social support [18-21] have a profound effect on health. Thus those without social networks are especially vulnerable [15]. Brown and Harris [22] underlined the importance of *social networks *for mental health, suggesting that coping with stressful events depends on whether a person has access to a social network as a protective factor.

Socioeconomic status (SES) and many of its components, such as *occupation *(a socioeconomic classification characteristic used in Sweden [23]), are also known to be associated with the mental health of immigrants from the FSU [24]. *Housing tenure *(owning or renting one's living quarters) is another important SES characteristic for studying a person's health. Residential areas with privately owned homes are occupied by people with good resources. The turnover of residents is less intensive in such areas and, consequently, there are more contacts between neighbors. "Such conditions give a freedom of action that is beneficial to health" [25].

*Cigarette smoking *has been shown to have a strong effect on health in various countries of the former Soviet Bloc in terms of mortality and morbidity [26-28]. However, smoking, like other unhealthy lifestyles, may be caused by the stressful psychological environment and social circumstances in these countries [19,29,30].

Acculturation – a complex social, cultural, and psychological process of adapting to a different society [31], is another determinant of mental health among immigrants from the FSU [10,32,33] and probably among immigrants from other countries of the former Soviet Bloc as well. An often used proxy of acculturation in quantitative studies is the mastery of the language of the host country. Greater mastery of the language has also been associated with better mental health in FSU immigrants [33].

The length of time since immigration has often been referred to as a health determinant and an indirect indicator of acculturation. Ritsner & Ponizovsky have shown that the acculturation of immigrants, and particularly the path of psychological distress connected with the process of acculturation, is not linear or U-shaped, but occurs in several phases [34]. Therefore, it is not necessarily true that the longer immigrants have spent in the host country, the better their acculturation and health will be.

However, *years spent in the host country *and *the year of immigration *may also indirectly represent the reason for migration, particularly for immigrants from the former Soviet Bloc to Sweden. While the late 1990s and the beginning of the new millennium brought many economic, labor and family reunion immigrants to Sweden from the countries of the former Soviet Bloc, the previous decades were generally characterized by the wave-immigration of asylum-seekers and political refugees escaping from the persecutions in the Soviet Bloc. The premigration experiences of life under comunist rule, defined by central planning, coercion, terror and communist nationalism [35] with the absence of basic human rights or freedoms and with one political party's domination in all aspects of economic and social life [36] could have affected immigrants' health. However, the studies do not agree on whether there is a relationship between health status and the communist regime *per se *(for a review, see [37]). However, just how the change from a communistic regime to democracy as a result of emigration negatively affects the mental health of Soviet immigrants has been the subject of debate [38]. *Years spent in the host country *also constitute the period of time when immigrants have been exposed to the post-migration factors in the host society. Migration to a country with different patterns of health and health behaviors, with greater economic satisfaction among its inhabitants and a different civil society might benefit the immigrant's health in time. On the other hand, such stressors of immigration as novelty, not feeling at home in the host country, sense of loss, and discrimination [10] in the host country might affect health negatively.

There are various possible pathways of causal influence between these, as well as other factors not mentioned here, and the mental health of immigrants from the former Soviet Bloc. The discussion of each one of them deserves consideration in its own right. Unfortunately, this is not possible in a short article. We therefore limited the scope of this study to the health determinants that have been particularly known to influence the health of persons born in these countries. Our intention in this study was to find out whether these determinants could explain the poorer mental health of immigrants from the former Soviet Bloc often reported in other studies [9,10,38-43]. Thus, the purposes of this study were: (1) to study whether the self-reported mental health (measured here as self-reported psychiatric illness and psychosomatic complaints) was poorer among persons born in the countries of the former Soviet Bloc currently living in Sweden than among Swedish-born persons and (2) to investigate whether the demographic, socioeconomic, and immigration-related characteristics of respondents could explain this.

## Methods

### Setting and sample

This study was based on eight pooled, cross-sectional random samples of permanent Swedish residents (including immigrants with permanent residence permits or Swedish citizenship). The samples consisted of men and women aged 25 to 84, of whom 385 were immigrants from Poland (n = 161), Eastern Europe (n = 164), and the FSU (n = 60) who arrived in Sweden after 1944. The information was collected by trained interviewers in face-to-face interviews as part of the Swedish Annual Level of Living Survey (SALLS) between 1994 and 2001.

The reliability of the survey questions was tested in other contexts in Swedish [44] and Latin American [45] subsamples and was found to be high. Moreover, the external validity of the self-reported severe or very severe long-standing illness was high. An overreporting frequency of 10% and underreporting of 20% was found [45,46].

Although ideally it would have been desirable to test the reliability of the questions in the immigrants from the former Soviet Bloc as well, this was not possible because the current study was undertaken 5 to 12 years after the original surveys (1994 to 2001) were made.

### Dependent variables

Self-reported mental health was defined in the present study as the *self-reported psychiatric illness *and *psychosomatic complaints*.

*Self-reported psychiatric illness *was derived from two questions:

1) "Have you any long-standing illness, after-effects of injury, disability, or other ailment? Name and describe in detail each ailment/illness." The coding group at Statistics Sweden assigned diagnoses according to ICD-9 (even after 1997) to everything the respondents had reported when answering this question. For the present study, all respondents with the diagnoses 290–319 were defined as having a self-reported long-standing psychiatric illness.

2) "Do you have anxiety?" (Yes, severe; Yes, slight; No). Those who answered "Yes, severe" and "Yes, slight" were regarded as having anxiety.

All persons who reported having any long-standing psychiatric illness (1) and/or anxiety (2) were categorized as having a *self-reported psychiatric illness*.

*Psychosomatic complaints *were derived from the questions:

(1) "Have you experienced any of the following during the last two weeks: (a) often feeling tired; (b) feeling tired during the day; (c) feeling tired in the evenings; (d) suffering from repetitive headaches or migraine; and/or (e) sleeping difficulties?" (Yes/No)

(2) "Have you (f) experienced aching in the shoulders or neck; (g) back pain, pain in the hips or sciatica; (h) pain or aching in the hands, elbows, legs, or knees?" (Yes, severe; Yes, slight; No). Those who answered "Yes, severe" *or *"Yes, slight" were considered to experience aching or pain.

Persons reporting three or more of the above complaints (a-h) were considered to have *psychosomatic complaints*.

### Independent variables

*Country of birth *was categorized into:

- "Sweden" (reference group);

- "Poland";

- "Other East European countries" – a composite of Hungary, Bulgaria, the Czech Republic, Slovakia, and Romania; and

- "The former Soviet Union" – a composite of all 15 former republics.

With the exception of the German Democratic Republic (GDR), all the countries once making up the Soviet Bloc were included in the study. The GDR was excluded because it has been coded as "Germany" together with the Federal Republic of Germany in the SALLS database for the majority of the years of the present study. This made it impossible to define people born specifically in the GDR for our 1994–2001 study period.

The official date of the creation of the Soviet Bloc is May 14, 1955, although Eastern Europe had *de facto *already come under Soviet control at the end of the Second World War. Consequently, we chose to include immigrants from the countries of the former Soviet Bloc who came to Sweden after 1944, rather than after its official date of creation. We have also included immigrants who arrived after its dissolution in 1989 because they had lived in the Soviet Bloc before that date.

Other independent variables were: *age *(the lower age limit was set at 25 when, presumably, the majority of young people start working and living on their own), *sex*, and *marital status *("married/cohabiting"/"single" with or without children under 18 years old). The *social network *index was based on the frequency of contacts with a close friend (from 1–6 days ago to more than 12 months ago) and the neighborly exchange of small services (from several times a week to never). The answers were ranked from 0 to 5 and the scores for these answers were added up (range: 0–10). Participants with a score of less than 3 were considered to have a "good" social network, others to have a "poor" social network. The *social network *variable measured as the frequency of contacts does not reflect the 'extensiveness' and 'resourcefulness' of these contacts; nevertheless, it represents the respondent's potential for obtaining support (emotional or material) if in need.

Two proxies of SES, *occupation *and *housing tenure*, were included as independent variables as well. *Occupational status *("higher and middle white-collar"; "lower white-collar", "self-employed", "student", or "blue-collar worker") was regarded as present or, in cases of current retirement/unemployment, previous work. *Housing tenure *referred to "owning"/"renting" one's home. The employment variable (employed/unemployed) was not included since it did not allow ordinary pensioners, early retirement pensioners, disability pensioners, or students to be taken into account.

One variable characterizing the lifestyle of respondents was included in the present analysis, i.e. *smoking *("Yes/No"). However, the choice of the lifestyle variable was determined by the limitation of our data, namely the incomplete data on other lifestyle characteristics.

The following variables were used in the additional analysis as migration-related variables: *language spoken at home *("Swedish"/"Other"), and *years in Sweden *("1–5", "6–10", "11–19", or "> 20"). *Year of immigration to Sweden *("1945–1949"; "1950–1959", "1960–1969", "1970–1979", "1980–1989", and "1990–1999") is presented in Table 3 as well, but it was not used in the additional analysis (Table 5). This variable highly correlated with *years in Sweden *and yielded almost the same result, however, with a lower fit of data.

**Table 3:** Descriptive Characteristics of the Study Population by Country of Birth (%) and Prevalence of Outcomes (%): SALLS, 1994–2001, Age 25–84

		Country of birth			
		
Variable	Level	Sweden	Eastern Europe		The former Soviet Union
				
			Poland	Other	
Sample size	N	35,459	161	164	60
Age, mean	Years	50.4	47.0	48.8	50.3
Sex	Female	51.1	68.9	52.4	58.3
	Male	48.9	31.1	47.6	41.7
Marital status	Married/Cohabiting	70.8	72.7	70.7	65.0
	Single	29.2	27.3	29.3	35.0
Social network	Good	81.4	78.9	78.1	65.0
	Poor	18.5	21.1	21.9	35.0
Occupation status	White-collar worker	32.4	40.4	34.2	40.0
	Lower white-collar worker	14.4	9.3	11.0	10.0
	Self-employed worker	10.4	5.0	8.5	5.0
	Student	2.8	5.6	6.1	8.3
	Blue-collar worker	40.0	39.7	40.2	36.7
Housing tenure	Ownership	71.0	51.6	51.2	48.3
	Renting	29.0	48.4	48.8	51.7
Smoking	No	80.2	70.2	77.4	76.7
	Yes	19.8	29.8	22.6	23.3
Language at home	Swedish	-	60.9	57.9	60.0
	Other	-	39.1	42.1	40.0
Years in Sweden	> 20	-	53.4	59.8	48.3
	11–19	-	32.3	21.3	15.0
	6–10	-	11.2	15.2	20.0
	1–5		3.1	3.7	16.7
Year of immigration to Sweden	1945–49	-	4.4	5.5	26.7
	1950–59	-	6.8	22.0	8.3
	1960–69	-	15.5	20.1	6.7
	1970–79	-	34.2	14.6	10.0
	1980–89	-	32.3	28.7	15.0
	1990–99	-	6.8	9.1	33.3
Self-reported psychiatric illness	Prevalence (binomial confidence interval)	17.8	34.8	32.9	21.7
		(17.4–18.2)	(27.5–42.7)	(25.8–40.7)	(12.1–34.2)
Psychosomatic complaints	Prevalence (binomial confidence interval)	42.0 (41.5–42.5)	64.6 (56.7–72.0)	51.8 (43.9–59.7)	46.7 (33.7–60.0)

Abbreviations: SALLS, Swedish Annual Level of Living Survey

**Table 5:** Association Between Country of Birth and Outcome Variables Adjusted for All Dependent Variables, Including the Migration-Related Variables *Language Spoken at home *and *Years in Sweden*: SALLS 1994–2001, Age 25–84 (Swedish-born respondents excluded)

	Country of birth	Eastern Europe		The former Soviet Union
			
		Poland	Other	
	Sample size (n)	161	164	60

Psychiatric illness				

Model 1a	OR (95% CI) Adjusted for all variables (without migration-related ones)	2.06 (1.00–4.24)	2.01 (0.98–4.13)	1 (Reference)
Model 2a	OR (95% CI) Adjusted for all variables + *Language at home*	2.11 (1.02–4.35)	2.05 (1.00–4.24)	1 (Reference)
Model 3a	OR (95% CI) Adjusted for all variables + *Years in Sweden*	1.92 (0.90–4.08)	1.96 (0.93–4.12)	1 (Reference)
Model 4a	OR (95% CI) adjusted for all variables, incl. both *Language at home *and *Years in Sweden*	1.94 (0.91–4.14)	1.97 (0.93–4.18)	1 (Reference)

Psychosomatic complaints				

Model 1b	OR (95% CI) Adjusted for all variables (without migration-related ones)	2.03 (1.10–3.78)	1.30 (0.71–2.38)	1 (Reference)
Model 2b	OR (95% CI) Adjusted for all variables + *Language at home*	2.04 (1.07–3.79)	1.29 (0.70–2.38)	1 (Reference)
Model 3b	OR (95% CI) Adjusted for all variables + *Years in Sweden*	1.67 (0.87–3.19)	1.12 (0.60–2.11)	1 (Reference)
Model 4b	OR (95% CI) adjusted for all variables, incl. both *Language at home *and *Years in Sweden*	1.65 (0.86–3.16)	1.10 (0.60–2.53)	1 (Reference)

Abbreviations: SALLS, Swedish Annual Level of Living Survey; OR, odds ratio; CI, confidence interval.

*Language spoken at home *represented mastery of language under the assumption that the general mastery of language partly depends on whether a person speaks Swedish at home (i.e. constantly, in contrast to immigrants who only use it at work or other public places).

*Years spent in Sweden *and *year of immigration to Sweden *were included in the study not as proxies of acculturation, but rather to indirectly control for reasons for migration in the defined sample.

### Statistical methods

The data were analyzed using the STATA software package [47]. Unconditional logistic regression [48] was applied to estimate the odds ratios (ORs) of poor self-reported health, and the results were presented with a 95% confidence interval (CI). First-order interactions between country of birth and independent variables (excluding migration-related ones) were tested. Full models included these independent variables analyzed jointly. An analysis including the migration-related variables was performed likewise on immigrant groups only (the FSU immigrants were used as a reference). The Hosmer and Lemeshow goodness-of-fit test [49] was used to assess the fit. All models showed a satisfactory fit (*p *> 0.05).

Ethical approval of the study was obtained from the Ethics Committee of the Karolinska Institute, Sweden.

## Results

The three groups of immigrant respondents differed slightly with respect to the descriptive characteristics (Table 3). The largest difference was seen in the year of immigration to Sweden, with immigrants from Poland arriving during 1970–1989, immigrants from the Eastern Europe during 1950–1969 and 1980–1989, whereas immigrants from the FSU mostly arrived right after the war and after 1990 (Table 3). It is noteworthy that the year of immigration to Sweden reflected the occurrence of major events in the countries of birth of these immigrants (Table 1). However, it did not fully mirror the arrival data presented in Table 2. Since the data in Table 2 and the data used for the analysis in the present study stemmed from the same source (Statistics Sweden), this slight discrepancy might be explained by the fact that the data in Table 2 were collected during one year (2001), and the data used for the analysis in the present study were pooled from eight years (1994–2001).

The personal characteristics (except the migration-related ones) were included in the logistic regression analysis as independent variables. The odds of reporting psychiatric illness and/or psychosomatic complaints varied in different categories of the independent variables. For example, women reported psychiatric illness and psychosomatic complaints almost twice as often as men. Similarly, respondents occupied with other than the white-color work, respondents who rented their homes, and those who smoked reported psychiatric illness and psychosomatic complaints more often than their counterparts. Those who were single reported psychiatric illness more often than those who were married/cohabiting, as did respondents with a poor social network, compared to those with a good social network. Most importantly, respondents who were born in Poland or other East European countries reported both psychiatric illness and psychosomatic complaints almost twice as often as the respondents born in Sweden. When all variables were included in the analysis simultaneously, the odds ratios changed marginally and remained as described above (Table 4).

**Table 4:** Odds Ratios [OR (95% CI)] for Self-Reporting Psychiatric Illness or Psychosomatic Complaints in the Study Population (simultaneous adjustment for all the independent variables, except migration-related ones): SALLS 1994–2001

Variable	Level	OR (CI 95%) Self-reported psychiatric illness	OR (CI 95%) Self-reported psychosomatic complaints
Country of birth	Sweden	1 (Reference)	1 (Reference)
	Poland	2.17 (1.55–3.03)	2.27 (1.63–3.15)
	Other East European countries	2.17 (1.54–3.02)	1.44 (1.05–1.96)
	The former Soviet Union	1.09 (0.58–2.05)	1.16 (0.69–1.93)
Age	25–44	1 (Reference)	1 (Reference)
	45–64	1.13 (1.06–1.20)	1.04 (0.99–1.10)
	65–84	1.14 (1.06–1.23)	0.88 (0.83–0.94)
Sex	Women	1 (Reference)	1 (Reference)
	Men	0.56 (0.52–0.59)	0.54 (0.52–0.57)
Marital status	Married/cohabiting	1 (Reference)	1 (Reference)
	Single	1.65 (1.56–1.76)	1.06 (1.01–1.11)
Social network	Good	1 (Reference)	1 (Reference)
	Poor	1.48 (1.38–1.59)	1.04 (0.98–1.10)
Occupation status	White-collar	1 (Reference)	1 (Reference)
	Lower white-collar	1.24 (1.14–1.35)	1.24 (1.16–1.33)
	Self-employed	1.09 (0.98–1.21)	1.37 (1.27–1.48)
	Students	1.47 (1.26–1.72)	1.18 (1.04–1.35)
	Blue-collar workers	1.21 (1.13–1.29)	1.52 (1.44–1.60)
Housing tenure	Ownership	1 (Reference)	1 (Reference)
	Renting	1.28 (1.20–1.36)	1.12 (1.07–1.18)
Smoking	No	1 (Reference)	1 (Reference)
	Yes	1.32 (1.24–1.41)	1.23 (1.16–1.30)

Abbreviations: SALLS, Swedish Annual Level of Living Survey; OR, odds ratio; CI, confidence interval.

These results suggest that there is an association between the outcomes and several of the independent variables, the association which could not be explained by other included variables. The association between the self-reporting of both psychiatric illness and psychosomatic complaints and the country of birth was the most profound: the odds ratios for reporting the outcomes were two times higher among persons born in Poland and other East European countries than among the respondents born in Sweden.

This association has been tested further. The FSU-born respondents were treated as a reference group, Swedish-born respondents were excluded (Table 5, Models 1a and 1b), and migration-related variables were added one-by-one into the analyses (Table 5, Models 2a, 2b and 3a, 3b). *Language at home *did not influence the odds of the outcomes, but *years in Sweden *(Model 3b) largely decreased the odds ratio of reporting psychosomatic complaints among immigrants from Poland and other East European countries. Thus it can be regarded as a confounder for psychosomatic complaints, but not for psychiatric illness (Model 3a).

When compared between themselves with both additional variables included simultaneously (Table 5, Models 4a and 4b), the immigrant groups showed the same pattern: both the respondents born in Poland and other East European countries had higher odds ratios than the respondents born in the former Soviet Union, irrespective of the outcome. However, the odds ratios were not significantly increased. This can partly be explained by the small sample size in the group of respondents born in the FSU.

To test the main results of the study, we conducted an analysis of hospital admissions based on data for all hospitalizations in the entire nation. We used the Cox regression to calculate the age- and sex-adjusted hazard ratios (HRs) for first hospital admissions due to mental disorders among immigrants from the former Soviet Bloc 25–84 years old, who arrived to Sweden after 1944 and lived in Sweden during 1994–2001. We found that both women and men born in Poland (HR = 1.43; CI = 1.32–1.56), as well as women born in other East European countries (HR = 1.15, CI = 1.01–1.31) had a higher hazard ratio of being hospitalized due to mental disorders than the Swedish-born women and men. However, persons born in the FSU, regardless of their sex, did not have a higher hazard ratio compared to Swedish-born persons (HR = 1.09, CI = 0.96–1.24).

## Discussion

Country of birth showed to have a profound influence on self-reporting of psychiatric illness and psychosomatic complaints among the immigrants from the countries of the former Soviet Bloc in Sweden. Other variables often mentioned in the literature on the health of immigrants from or populations inhabiting the countries of the former Soviet Bloc (i.e. demographic and socioeconomic variables) influenced the self-reported mental health, but could not explain the association between the country of birth and the outcomes.

This is in conformity with the small number of other studies showing that *country of birth *has a more profound influence on mental health than age [50] and than socioeconomic status [51,52] among immigrants from some East European countries and Russia in Sweden.

The influence of the country of birth on the self-reported mental health differed among the three immigrant groups in the present study. Immigrants from Poland and other countries of Eastern Europe had poorer self-reported mental health with, in general, twofold higher odds of reporting psychiatric illness and psychosomatic complaints than persons born in Sweden. This could not be explained by the demographic or socioeconomic variables. However, immigrants from the FSU had odds of reporting psychiatric illness and psychosomatic complaints similar to those of the Swedish-born reference group. This finding was also supported by the analysis of the of hospital admissions due to mental disorders.

The finding of higher odds for poor self-reported mental health among immigrants from Poland and other East European countries in the present study agrees with the studies of self-reports and of hospital admissions from Sweden, Britain, and the US and with other cross-sectional and longitudinal studies, as well as studies on war and on political refugees [39-42]. It is difficult to say whether the results depend on premigration experiences in the countries of birth which determined the health of immigrants from these countries or whether the post-migration experiences in Sweden, particularly difficulties of acculturation in the new country usually experienced by immigrants, defined the result. Probably both. However, the aim of this study was not to clarify that matter and the question remains to be answered.

The finding that immigrants from the FSU had similar odds of poor self-reported mental health to those of the host population does not agree with studies conducted elsewhere (the vast majority of the earlier studies have been made in the US and Israel) [9,10,38,43]. A plausible explanation for this discrepancy is that the mental health of the FSU immigrants in Sweden might differ from that of their countrymen in other countries due to different migration trends and various factors in the host countries. Moreover, many other international studies have focused on specific ethnic groups from the FSU (e.g., only Russians or Russian Jews), geographically defined communities, psychiatric patients, or other selected groups that have higher risks of mental disease [9,33,53]. In contrast, the present study included immigrants from various FSU republics living anywhere in Sweden. This was ensured by drawing our sample from the national representative sample of permanent Swedish residents recorded in the Register of the Total Population and interviewed in SALLS between 1994 and 2001.

We can only speculate as to why immigrants from the FSU did not have higher odds of poor self-reported mental health than the Swedish-born host population and had lower odds than immigrants from the other former Soviet Bloc countries (nonsignificant). It is possible that persons born in the FSU are afraid of stigmatization, have a more negative attitude toward mental disorders and their treatments (as a legacy of the Soviet abuse of mental diagnoses and medical services for political purposes in the past) and are inclined not to report poor mental health or seek help for mental disorders. The lower odds for psychosomatic complaints among immigrants from the FSU compared to immigrants from Poland might also be explained by the fact that persons born in the FSU are inclined to hide their private feelings [54], to be ashamed of their physical and psychological problems, and tend not to share them with others [55]. However, Hitch and Rack [41] give another explanation, suggesting that weaker mutual support in Polish communities than in Ukrainian ones, social seclusion from the host population and the fellow nationals might partly explain the higher incidence of first admissions to psychiatric hospitals among Polish immigrants than Ukrainian immigrants.

Another possible explanation of the different odds for poor self-reported mental health among immigrants from the former Soviet Bloc is better mental health among persons born in the FSU than among those born in the other former Soviet Bloc countries. According to the World Health Organization (Health for All Database) in 2002, the prevalence of mental disorders in the FSU (2.8%) was lower than in the former Czechoslovakia (3.5%) and Poland (3.2%).

It is very difficult, however, to compare rates of self-reported mental health since the self-administered screening instruments in psychiatry show different cut-off points in different cultures. For example, *psychosomatic complaint *is a culturally sensitive variable because representatives of every nation have their specific way of somatizing or expressing their ill-health. Inhabitants in or immigrants from different countries communicate their health, particularly mental health, differently [53,55-57]. The fact that the *psychiatric illness *variable was self-reported and not clinically validated was the most important limitation of the study. Self-reported health is a reliable indicator of health [5,6], preterm mortality [58-61], and health care need [62,63]. However, it has not been confirmed whether self-reported mental health is also a reliable indicator. It is likely that the prevalence of poor self-reported mental health is undeerestimated in the present study since the respondents are generally probably less willing to report a psychiatric disorder than a somatic one.

Nevertheless, the additional testing of this variable by performing an analysis of the hospital admission data for mental disorders in the same population supported the results of the present study, confirming our assumption that self-reported mental health may be a good indicator of mental health. Moreover, self-reported mental health is associated with mortality as self-reported health is [64,65] and we therefore believe that self-reported mental health is a valuable indicator in population health monitoring.

Another limitation of the present study is that the immigrant groups focused on probably lacked homogeneity not only between themselves with regard to self-reported mental health and other characteristics (including historical backgrounds, religion, language, and geography), but also within themselves. For example, the three Baltic FSU republics have joined the European Union and differ from the other FSU republics in many other respects. Therefore, it is difficult to say whether the results of the present study are fully applicable to immigrants from these now independent countries when considered separately from the whole FSU; thus this applies to the East European countries. Nevertheless, immigrants with different backgrounds had to be combined in larger categories ("Other East European countries" and "The former Soviet Union") to achieve sufficient statistical power and to conduct the analyses in the present study. Poland was categorized separately due to a large number of Polish immigrants in Sweden. The FSU was categorized separately due to differences in the economy and health of the inhabitants of the FSU compared to other East European countries [14].

The limitations of the cross-sectional design of the present study prevented us from tracing any causal pathways between the migration of people from the former Soviet Bloc and self-reported mental health, and we could only speculate about the determining mechanisms behind the reporting of psychiatric illness and psychosomatic complaints. A lack of data on alcohol consumption was also a limitation for a study on self-reported mental health, although an adjustment of outcomes was made for smoking. The questionnaire was not specifically designed for the present study and we had to use the available data rather than collect our own. Additionally, it was not possible to perform a clinical evaluation to test the reliability of the instruments because the design of the national survey does not include a clinical examination or a clinical instrument.

Finally, the sample size, especially of immigrants from the FSU, was small and possibly affected the significance of the analysis. However, in spite of its small size, it included immigrants from the various former Soviet republics, and the proportion of respondents was representative of all 25–84-year-old FSU immigrants who arrived in Sweden after 1944. The weighted mean population from our sample was similar to the average number of persons of the same age and country of birth living in Sweden registered by Statistics Sweden during 1994–2001 (not shown).

The strengths of the extensive SALLS data balanced the limitations. SALLS is performed annually by Statistics Sweden and is designed to obtain information about the Swedish living conditions of the Swedish population. Statistics Sweden is a governmental statistical bureau with a long tradition of carrying out national surveys and analyzing the collected data. The data collected by Statistics Sweden is used in multiple studies and for the national statistical reports. The reliability of the survey questions is high and the nonresponse is rather low. The nonresponse rate in SALLS during 1994–2001 was about 20%, mostly due to refusal to take part. In the earlier samples it was found that those who refused to participate (2/3 of nonrespondents) had the same mortality rate as the respondents. Regretfully, it was impossible to estimate the particular nonresponse among the immigrants in this study because the sample in SALLS is not chosen according to the country of birth.

A more detailed description of the SALLS can be found elsewhere [66].

## Conclusion

According to the findings of the present study, the influence of country of birth on the self-reported mental health was profound and could not be explained by other health determinants, such as age, sex, marital status, social network, SES, or smoking.

The self-reported mental health differed, however, among immigrants from the different former Soviet Bloc countries. Unlike the immigrants from the FSU, immigrants from Poland and other East European countries generally had a twofold higher odds ratio for reporting psychiatric illness and psychosomatic complaints than adults born in Sweden after adjusting for demographic and socioeconomic variables. An adjustment for migration-related variables changed it only to the limited extent.

These findings and prior awareness of the health of immigrants from countries focused on in the present study should be acknowledged, particularly with the continuing expansion of the European Union.

## Competing interests

The author(s) declare that they have no competing interests.

## Authors' contributions

All three authors have made substantial contributions to the present study. All the authors read and approved the final version of the manuscript.

YB performed the analyses and the literature review and was mainly responsible for the writing of the manuscript.

SEJ participated in the design of the study and coordinated the statistical analysis and helped to revise an earlier version of the manuscript.

JS participated in the design and coordination of the study and helped to draft the manuscript.

## Pre-publication history

The pre-publication history for this paper can be accessed here:


